# A cautionary signal from the Red Sea on the impact of increased dust activity on marine microbiota

**DOI:** 10.1186/s12864-022-08485-w

**Published:** 2022-04-07

**Authors:** Hayedeh Behzad, Hajime Ohyanagi, Badr Alharbi, Martin Ibarra, Mohammed Alarawi, Yoshimoto Saito, Carlos M. Duarte, Vladimir Bajic, Katsuhiko Mineta, Takashi Gojobori

**Affiliations:** 1 Computational Bioscience Research Center (CBRC), King Abdullah University of Science and Technology (KAUST), Thuwal, 23955-6900 Saudi Arabia; 2 Biological and Environmental Sciences and Engineering Division (BESE), King Abdullah University of Science and Technology (KAUST), Thuwal, 23955-6900 Saudi Arabia; 3 National Centre for Environmental Technology, King Abdulaziz City for Science and Technology, Riyadh, 11442 Saudi Arabia; 4 Marine Open Innovation (MaOI) Institute, Shizuoka, 424-0922 Japan; 5 Red Sea Research Center (RSRC), King Abdullah University of Science and Technology (KAUST), Thuwal, 23955-6900 Saudi Arabia; 6 Computer, Electrical and Mathematical Sciences and Engineering Division (CEMSE), King Abdullah University of Science and Technology (KAUST), Thuwal, 23955-6900 Saudi Arabia

**Keywords:** Global climate changes, Increased dust emission, Marine microbiota, Metagenomics

## Abstract

**Background:**

Global climate change together with growing desertification is leading to increased dust emissions to the atmosphere, drawing attention to possible impacts on marine ecosystems receiving dust deposition. Since microorganisms play important roles in maintaining marine homeostasis through nutrient cycling and carbon flow, detrimental changes in the composition of marine microbiota in response to increased dust input could negatively impact marine health, particularly so in seas located within the Global Dust Belt. Due to its strategic location between two deserts and unique characteristics, the Red Sea provides an attractive semi-enclosed “megacosm” to examine the impacts of large dust deposition on the vastly diverse microbiota in its exceptionally warm oligotrophic waters.

**Results:**

We used culture-independent metagenomic approaches to assess temporal changes in the Red Sea microbiota in response to two severe sandstorms, one originated in the Nubian Desert in the summer 2016 and a second one originated in the Libyan Desert in the spring 2017. Despite differences in sandstorm origin and meteorological conditions, both sandstorms shifted bacterial and Archaeal groups in a similar mode. In particular, the relative abundance of autotrophic bacteria declined while those of heterotrophic bacteria, particularly Bacteroidetes, and Archaea increased. The changes peaked within six days from the start of sandstorms, and the community recovered the original assemblage within one month.

**Conclusion:**

Our results suggest that increased dust emission with expanding desertification could lead to undesirable impacts in ocean function, enhancing heterotrophic processes while reducing autotrophic ones, thereby affecting the marine food web in seas receiving dust deposition.

**Supplementary Information:**

The online version contains supplementary material available at 10.1186/s12864-022-08485-w.

## Background

Large areas of lands around the world are faced with increased dryness, desertification, and dust emission and deposition due to global climate changes and anthropogenic causes [1–8]. The majority of dusts originate from hyper-arid and arid drylands, in particular the Sahara Deserts in Africa, the Gobi and Taklamakan Deserts in Asia, and the Arabian Deserts in the Middle East [9]. Drylands cover over 40% of the earth’s landmass, and include varying types of biomes from hyper-arid deserts to arid, semi-arid, and dry sub-humid arable lands, woodlands, and savannahs [10]. Drylands have expanded in the last half of the century, and simulation models under a high greenhouse gas emission scenario (RCP8.5) project that these regions could see a further increase of 5.8 × 10^6^ km^2^ or 10% by the end of the twenty-first century [10–12]. Concomitant with their expansion, drylands have become increasingly more susceptible to desertification due to global climate changes and anthropogenic activities [8, 11]. The current rate of land desertification is substantially greater than expected based on historic rates, with an approximately 12 × 10^6^ hectares of arable lands lost annually due to drought and desertification [13]. An important consequence of expanding desertification is increased emission of dust into the atmosphere and deposition in receiving ecosystems [4, 14]. The majority of these dusts are deposited in marine environments, which cover over two-thirds of the earth’s surface area [15]. Oceans are gatekeepers of the planet’s health and integral to the global carbon cycle [16]; therefore, understanding the impact of dust on marine environments could provide a window into global ecosystem stability.

Marine microbiota plays important functions in the biogeochemical cycling of nutrients in the ocean and the global carbon cycle, and long-term perturbations in their community composition and function could potentially be detrimental to marine ecosystem balance [16–18]. The short-term impact of dust on marine microbiome was examined previously in microcosm and mesocosm settings by a number of experimental [19–22] and modelling [23] studies, but a major caveat in previous research is the paucity of in situ field experiments. In particular, time-series analysis of the impact of natural sandstorms on microbial community composition in open marine environments is lacking due to their extraordinary demand on resources [24], the limited repeatability and reproducibility of *in situ* data [25, 26], and the unpredictability of extreme weather events such as sandstorm. Yet, *in situ*, event driven studies could produce a reliable representation of ocean responses to dust deposition, providing an essential validation to inferences derived from experiments and models, which simplify complexities [24, 27, 28], and thereby deliver needed predictive power on the responses of marine plankton communities to dust inputs.

The Red Sea is the world’s northernmost tropical sea located between two of the world’s largest deserts, the Sahara Deserts in Africa and the Arabian Deserts in the Middle East, which inject substantial quantities of dust into its exceptionally warm oligotrophic waters [29] during strong sandstorm events that hit the region on annual basis [30]. The Red Sea is a semi-enclosed environment connected to the world’s oceans only through a shallow strait of Bab Al-Mandeb (310 m deep) in the south through the Arabian Sea and a shallower Suez Canal (25 m deep) in the north through the Mediterranean Sea [31], which significantly limits exchanges with the ocean. A major dependency on dust as an external source of nutrients, lack of river inflow, minimal annual wet precipitations, limited seasonal temperature fluctuations, and the limited connection to the world’s oceans make the Red Sea a model environment to examine the *in situ* impact of increased dust activities on the vastly diverse microbiota present in its exceptionally warm hyper-saline waters [32, 33].

We examined the impact of two major sandstorms that swept the Red Sea region, one occurring in the summer 2016 and the other at the start of spring 2017. Specifically, we addressed the questions, 1) do sandstorms impact the composition of Red Sea microbial communities? and 2) if so, how persistent are these effects? This study investigates the temporal impact of natural sandstorms on microbial community composition in an *in situ* open marine setting, using the metagenomic approach.

## Results

### Back trajectories and environmental metadata

**Fig. 1:**
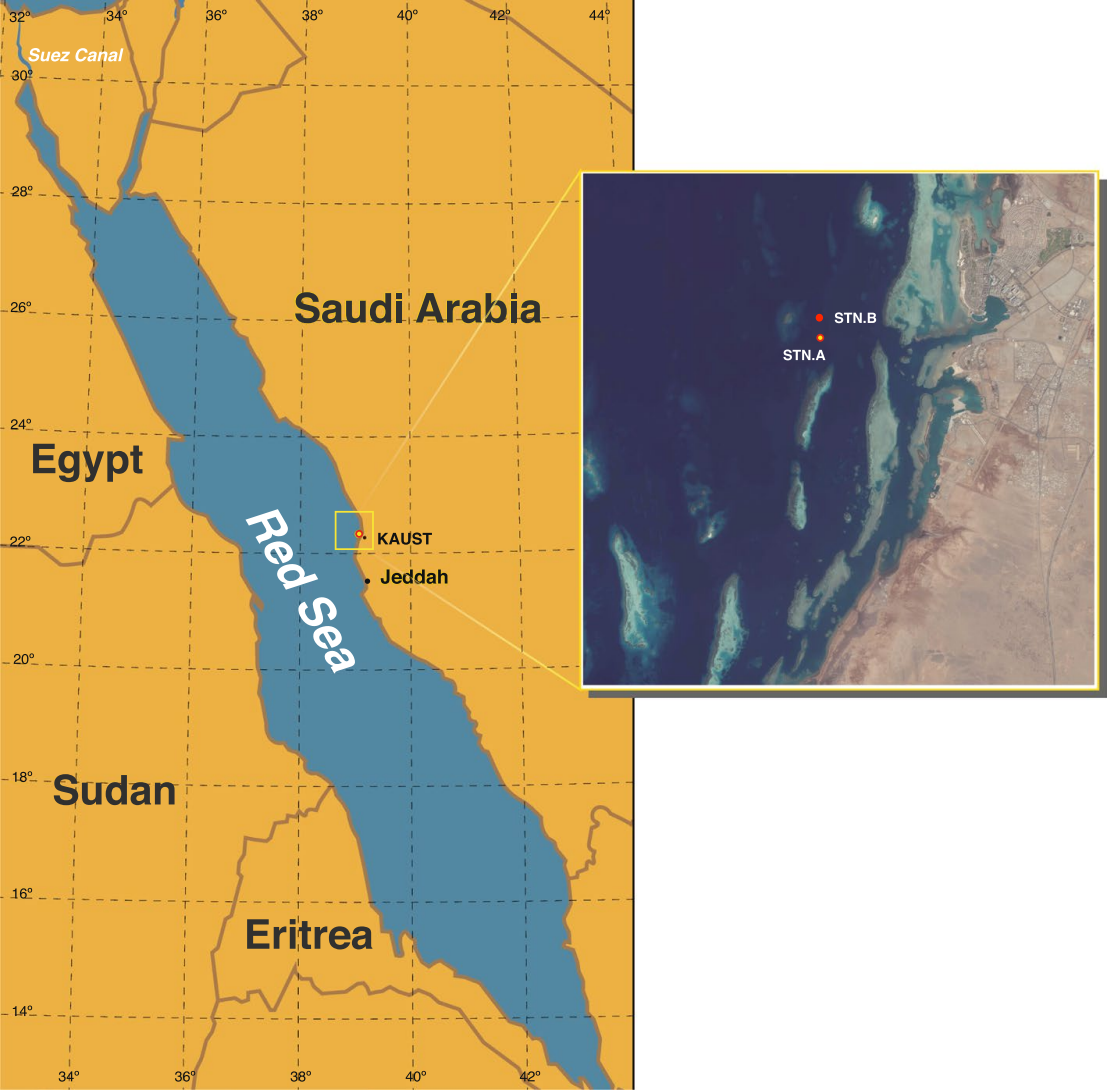
The 2016 and 2017 sampling locations. **a** Map drawing showing the sampling location (Red dot) in the Red Sea. **b** Magnified image of the white squared area in **a** showing the two sampling locations (STN. A and STN. B) in the central east coast of the Red Sea near KAUST (King Abdullah University of Science and Technology). The two sampling locations were approximately 1 km apart at coordinates 22° 17.988’N, 39° 03.427’E and 22° 18.549’N, 39° 03.480’E, respectively

**Fig. 2:**
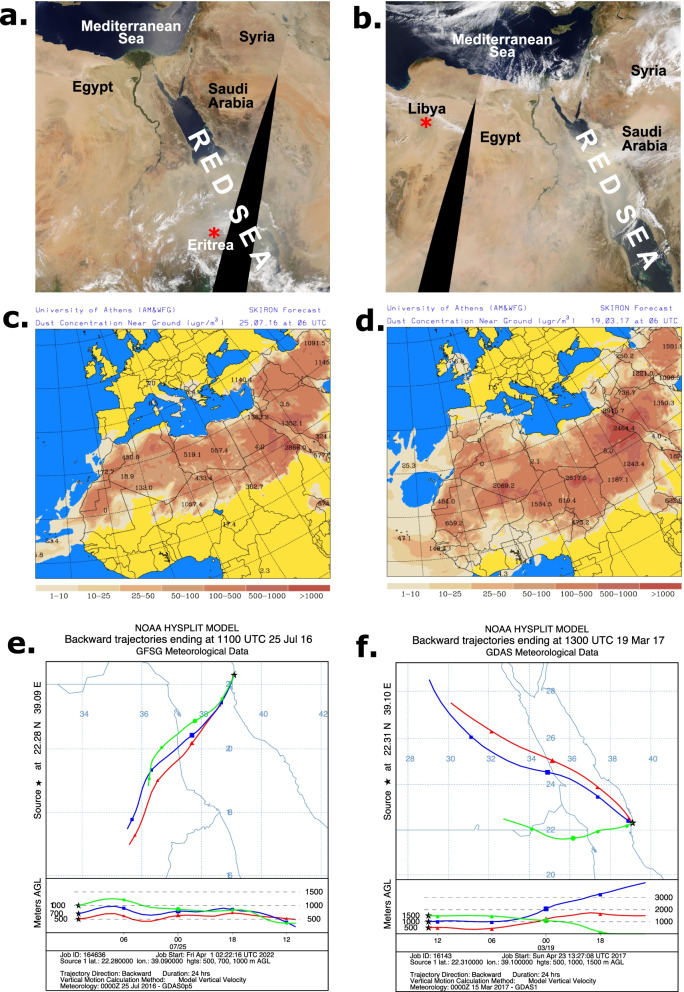
Dust activity maps and trajectory models. **a-b** NASA MODIS satellite images for **a** the summer 2016 and **b** the spring 2017 sandstorms. **c-d** The respective SKIRON weather forecast model showing the predicted dust concentration over the Red Sea during the sandstorm dates in **a-b** (obtained from the University of Athens). **e–f** The NOAA HYSPLIT back trajectories according to the direction of air parcels arriving at the vertical height of 500 m, 1000 m, and 1500 m above ground level, showing the origin of sandstorms in **a-b** respectively

Figure 1 shows the two sampling locations (STN. A and STN. B) in the central east coast of the Red Sea. Dust maps and trajectories for the summer 2016 and spring 2017 sandstorms are depicted in Fig. 2. Satellite data from the NASA Moderate Resolution Imaging Spectroradiometer (MODIS) in Fig. 2a-b demonstrate that the dust generated by these two sandstorms engulfed the entire width of the Red Sea in central-southern and central-northern regions, respectively. The SKIRON forecasting model (developed at the University of Athens) confirmed the presence of high concentrations of dust over the Red Sea during the two sandstorm events (Fig. 2c-d). The Hybrid Single-Particle Lagrangian Integrated Trajectory model (HYSPLIT) based back trajectory analyses pointed at the Nubian and Libyan Deserts on eastern and northern-eastern regions of the Sahara as origins of the 2016 and 2017 sandstorms, respectively (Fig. 2e-f). These sandstorms caused a sustained reduction in visibility, particularly so in the summer 2017 sandstorm, due to the higher concentrations of particulate matters with a diameter of 10 µm or less (PM_10_) in the air (See Table S1, Additional File 1).

### Illumina MiSeq 16S rRNA amplicon reads

Table S2 (Additional File 1) shows the average number of 16S rRNA amplicon reads before and after trimming, merging, filtering, and removal of chimeras with the final quality reads of 245,812 ± 9,838 and 222,601 ± 8,599 and average read size of 444 ± 0.2201 and 443 ± 0.2415 bp for the 2016 and 2017 samples, respectively. To ensure an even sequencing depth across all samples, reads were rarefied to 100,000 reads per sample prior to data analysis. The average number of reads and their sizes did not appear to be significantly different between the samples collected during the 2016 and 2017 sampling events (See Table S2, Additional File 1).

### Impact of sandstorms on richness and diversity of the Red Sea microbiota

**Fig. 3:**
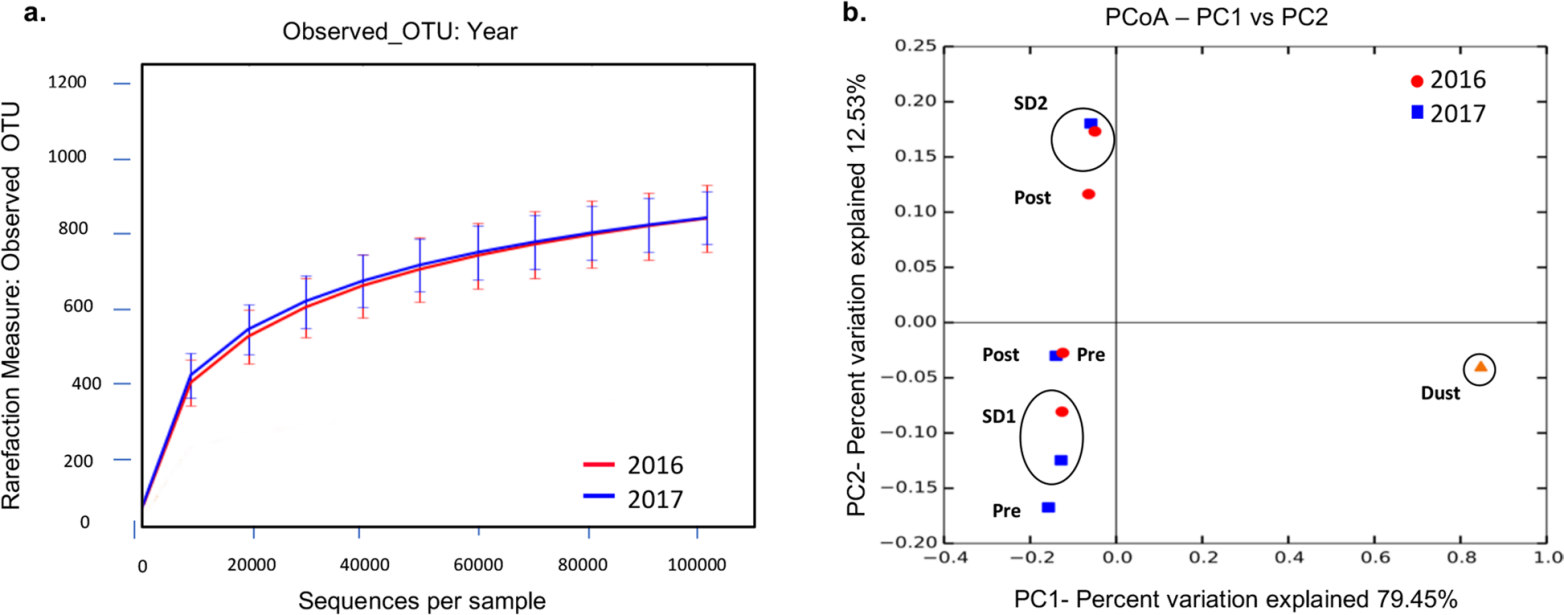
Richness and diversity of the Red Sea microbiota during the 2016 and 2017 sampling events. **a** The Rarefaction curve showing the average number of observed OTUs per sequences with the standard errors of the means for all the data from the 2016 and 2017 sampling events. **b** PCoA based on the weighted UniFrac distance matrix for the 2016 (red circles) and 2017 (blue squares) sampling events. Samples were averaged based on date (four dates per year). **Pre** (approximately one month prior to sandstorms; **SD1** (2–3 days after sandstorms); **SD2** (six days following sandstorms); **Post** (approximately one month after sandstorms). The Dust sample is from the 2017 (March 20^th^) sandstorm event

**Fig. 4:**
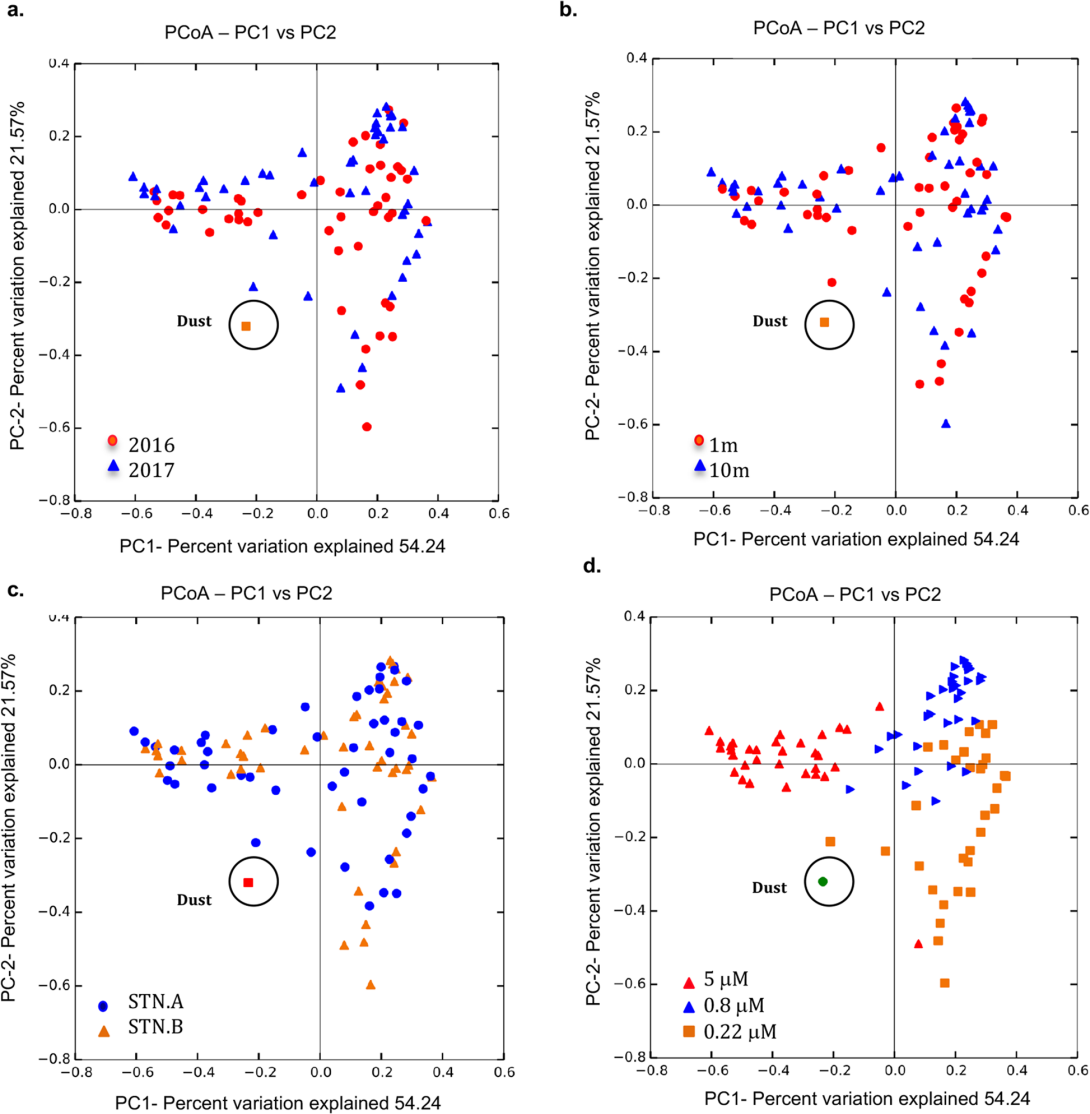
PCoA based on the weighted UniFrac distance matrix. Data were plotted according to sampling year (**a**) depths (**b**) stations (**c**) and pore sizes (**d**) for all the samples analyzed. Each point represents individual samples from different depth (1 m and 10 m), stations (STN.A and STN.B), and size fractions (5, 0.8, and 0.22micron filters) during the summer 2016 and spring 2017 sampling events. The circled point in each plot represents dust sample (Dust) collected from air during the 2017 (March 20^th^) sandstorm

The average richness, alpha diversity, and community structure of prokaryotes in the Red Sea surface waters were similar during the summer 2016 and the spring 2017 sampling events (Figs. 3a, 4a; Table S3a, Additional File 1), with a similar community composition at different sampling depth (1 m and 10 m) and location (STN.A and STA.B) [Fig. 4b-c]. Microbial diversity (Shannon and Wilson alpha diversity indices) increased within six days following each sandstorm, as shown in Table S3b, Additional File 1. The Principal Coordinate Analysis (PCoA) of weighted UniFrac distance matrix, used to compare alpha-diversity across different time points and sandstorm events, showed that six days following sandstorms, the abundance and composition of Operational Taxonomic Units (OTUs) in the Red Sea were surprisingly similar despite differences in sandstorm origin and associated meteorological conditions for the two events (Fig. 3b).

### Impact on microbial community composition

**Fig. 5:**
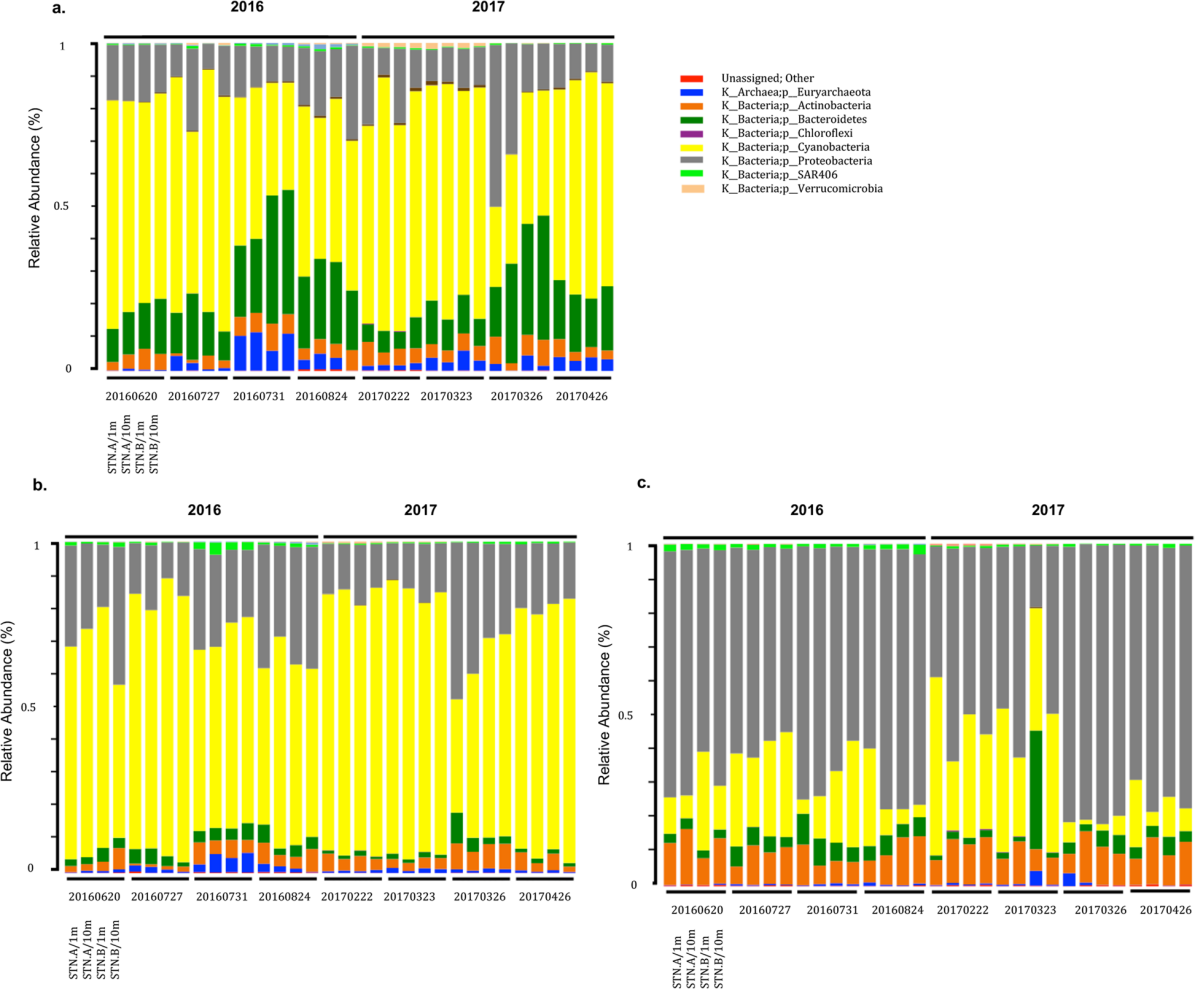
Percent relative abundance of bacteria and Archaea, at phylum level, in the Red Sea surface waters during the 2016 and 2017 sampling events. Graphs show changes in the most abundant OTUs on **a** 5micron filters; **b** 0.8micron filters; and **c** 0.22micron filters. For each time point, the data from 2 depth (1 m, 10 m) and 2 stations (STN.A and STN.B) were graphed side-by-side, as shown on the left lower corner of each graph

**Fig. 6:**
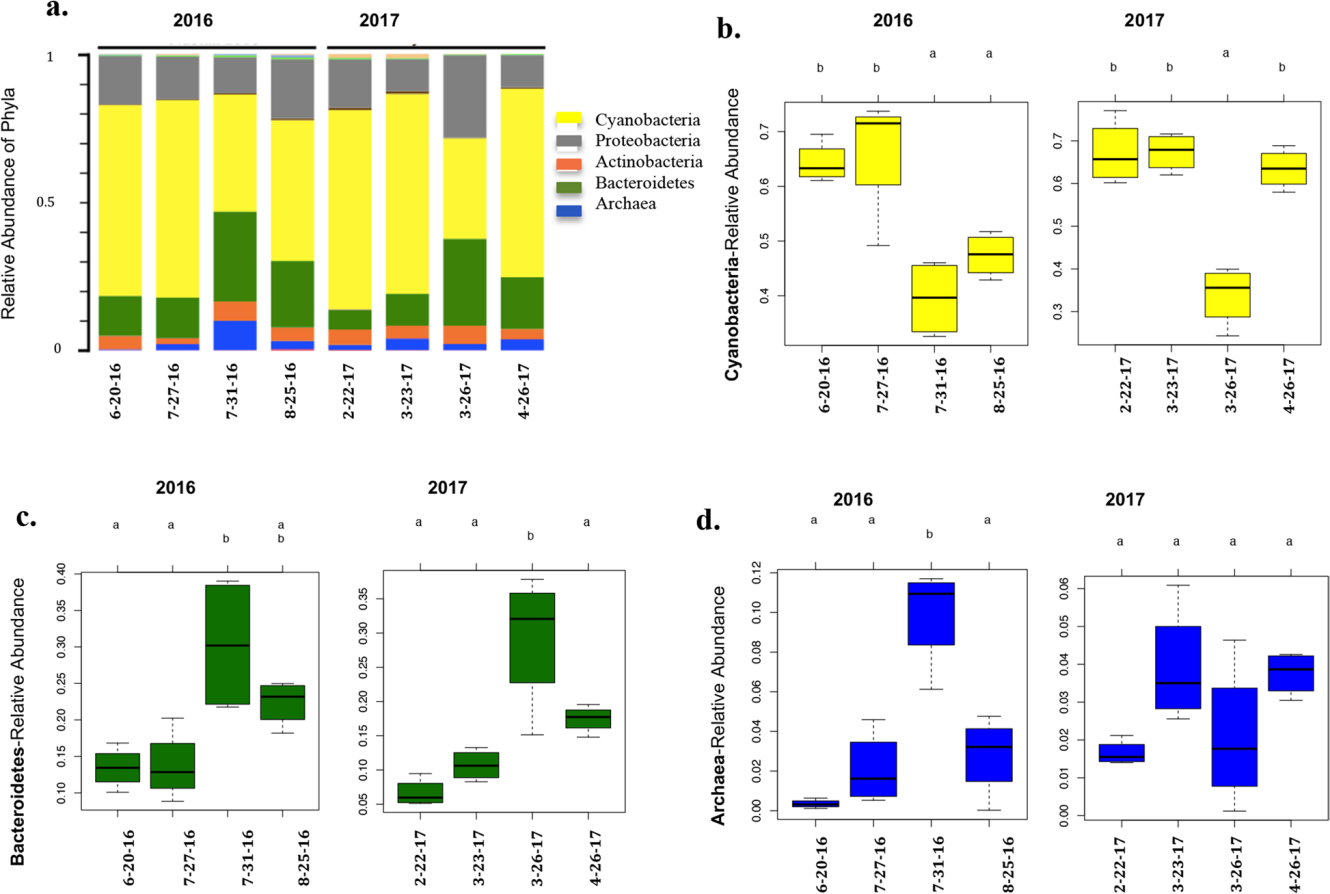
Taxonomic changes in microbial phyla in the Red Sea surface waters in response to the 2016 and 2017 sandstorms. **a** Bar graph shows changes in the average relative abundance of the most abundant Phyla present on 5micron filters, during the 2016 (left) and 2017 (right) sampling events. Box plots demonstrate the average relative abundance of the most affected phyla, as follow: **b** Cyanobacteria; **c** Bacteroidetes; **d** Archaea. Turkey’s HSD test was used to analyze significant differences between time points, where different alphabetical letters on top of each graph denotes significant differences between the time points (*p* < 0.05). The dates under each bar, from left to right, represent: 1st bar (approximately one month before sandstorm); 2nd bar (2–3 days following sandstorm); 3rd bar (six days following sandstorm); 4th bar (approximately one month following sandstorm)

The dominant microbial phyla in the Red Sea surface water were similar during the 2016 and 2017 sampling events, and included: Cyanobacteria, Proteobacteria, Actinobacteria, Bacteroidetes, and the Archaeal phyla, Euryarchaeota (Fig. 5). Both sandstorms consistently changed the relative abundance of similar groups of autotrophic vs. heterotrophic microorganisms. The relative abundance of Cyanobacteria, the main autotrophic bacterial phyla in the Red Sea surface waters, declined significantly within six days following sandstorms, parallel to a significant increase in the relative abundance of heterotrophic microorganisms, mainly those belonging to the phylum Bacteroidetes and Euryarchaeota. The most significant changes were observed on the microorganisms retained on 5 µm filters (Fig. 6), including filamentous, planktonic, and/or particle associated microorganisms (see Material and Methods on serial filtration), although moderately similar trends were also observed on microorganisms retained in the 0.8 µm (Fig. S1, Additional File 1) and 0.22 µm filters (Fig. S2, Additional File 1). The majority of these changes reverted back to their pre-sandstorm levels within one month following sandstorms (Fig. 6; Figs. S1-S2, Additional File 1), suggesting that the perturbation of sandstorms elapsed over a few weeks.

## Discussion

In this study, we examined temporal changes in the Red Sea microbial community composition, *in situ*, in response to two severe sandstorms that affected the region, one in the summer of 2016 and the other in the spring of 2017, both originating from African Deserts. Ours results demonstrate that regardless of differences in dust origin and time, sandstorms consistently decreased the relative abundance of autotrophic Cyanobacteria while increasing the relative abundance of heterotrophic bacteria and Archaea. The majority of these changes was temporary with the microbial community recovering their original assemblage within one-month following sandstorms. To the best of our knowledge, this is the first *in situ* study examining temporal shifts in marine microbial community composition on-site in the Red Sea in response to natural sandstorm events.

The decline in Cyanobacteria in response to sandstorms could be due to a number of factors including increases in grazing activities and viral lysis, as well as increased exposure to dust-delivered anthropogenic pollution. In agreement with our observations, a dust amended bioassay experiment in the central Atlantic Ocean demonstrated that Sahara dust had an overall negative impact on Cyanobacteria with a substantial reduction in primary productivity, although picoeukaryotes responded positively to dust addition [21]. Similarly, in dust amended studies conducted in the tropical Northeast Atlantic waters, Sahara dust leachates impaired the metabolic activity of *Prochlorococcus*, the major Cyanobacteria in the waters [34]. In a dust bioassay experiment in the Northern Red Sea surface waters, the high anthropogenic Cu^2+^ contents of Saharan dust aerosols were shown to be toxic to *Synechococcus* [20]*.* The Copper level in the Red Sea water was either undetectable or unchanged during the two sandstorm events (Table S4, Additional File 1). Desert dust aerosols might also carry other material that could kill cyanobacteria including, for example, viral phages [35]. A substantial increase in dissolved organic carbon following lysis of Cyanobacteria and other autotrophic microorganisms could enhance nutrient availability in the oligotrophic waters of the Red Sea, and subsequently increase the propagation of heterotrophic bacterioplanktons.

Consistent with this, we found a significant increase in the relative abundance of OTUs belonging to Bacteroidetes in the Red Sea surface waters in response to sandstorms. Bacteroidetes are generally one of the most abundance heterotrophic bacterial phyla in marine environments [36, 37], accounting for a major fraction of the bacterioplankton community, particularly in coastal regions where they constitute between 10%-30% of total bacterial counts [38]. Bacteroidetes can particularly take advantage of the newly available complex food sources following lysis of cyanobacteria and increase their abundance, since they have a host of enzymes and transporters for degrading complex polysaccharides and proteins [39, 40]. Bacteroidetes abundance was previously shown to increase with a rise in the input of high molecular weight dissolved organic matter (DOM) [41, 42], for instance during phytoplankton blooms in surface coastal waters, helping degrade the bloom-associated exopolymer particles [43–45]. Bacteroidetes can grow on polymeric substances and algal cells by utilizing organic polymers and algal metabolites as carbon and energy sources [46, 47]. They have gliding mobility and attachment capabilities and are generally found attached to particles and detritus material [40], which is in agreement with our findings in the Red Sea, where Bacteroidetes were present at highest relative abundance on 5micron filters, potentially attached to planktonic communities, particles, or complex detritus matters. Through attachment to complex organic matters, Bacteroidetes are able to directly access food and degrade complex carbohydrates and proteins into Low molecular weight DOM, which can then become accessible by other microorganisms, within the microbial loop, that have preference for less complex organic matters.

In addition to their impact on planktonic bacterial communities, sandstorms significantly increased the relative abundance of Archaea in the Red Sea. Like marine bacteria, Archaea play important roles in global ocean carbon and nitrogen cycles [48]. Euryarchaeota of the Marine Group II Archaea were the main Archaea found in the surface waters, the majority of which were detected on the large and medium size filters (5 micron and 8 micron), likely bound to particles and other planktonic communities. The Group II marine Archaea are heterotrophic/photoheterotrophic microorganisms present in ocean surface waters, bound to particles, and utilizing proteins and lipids polymers [49]. In agreement with our findings, Tan et al. found overrepresentation of Archaea in the metatranscriptomes obtained from dust addition studies in the Western North Pacific Ocean mesocosms [50]; among bacterial transcripts, those related to Bacteroidetes and Proteobacteria increased, while transcripts related to Cyanobacteria decreased dramatically [50]. Wells et al. found that terrestrial labile organic matters significantly impacted coastal Archaeal abundance in the Mackenzie River-influenced Beaufort Sea [51]; and the hike in the abundance of Group II Archaea in the Arctic Ocean was linked to the increased availability of terrestrially derived nutrients from the surrounding landmass [51]. A time series study conducted by Murray et al. demonstrated that “intermittent” blooms of Group II marine Archaea in Santa Barbara Channels coincided with decreased chlorophyll a concentration [52]. It is possible that, in our study, increased nutrient availability upon Cyanobacterial lysis and the terrestrial nutrient availability contributed to the observed increase in Archaeal relative abundance following sandstorm.

Whereas the microbial response patterns were consistent for both sandstorms, the mechanism(s) involved in microbiota restructuring in response to sandstorm cannot be resolved from our data. A number of potential mechanisms may be hypothesized, including the impact of dust nutrients, pollution, and exogenous dust-borne microorganisms [8], including lytic cyanophages, on the receiving Red Sea community.

Our results suggest that an increase in the frequency and input of dusts, associated with expanding desertification, might lead to irreversible changes in marine environments, through modulation of microbial community composition. A significant decrease in autotrophic bacteria with a concomitant increase in heterotrophic microorganisms could affect carbon flow to the marine food web and the recycling of nutrients, negatively impacting marine homeostasis. Addressing these concerns requires further monitoring of desertification, improved tracking of dust movements, along with expanded *in situ*, event driven, “megacosm” studies in the receiving seas to understand the causal relationships between dusts and marine health before potential tipping-points are reached.

## Conclusion

The increase in extreme meteorological events such as sandstorms could have adverse impacts on marine health through changes in its microbial community dynamic. In this study we have demonstrated the temporal impact of increased dust activities on marine microbiota in open waters in response to natural sandstorm events, using the high through metagenomic approach. Our data indicate that sandstorms changed the relative abundance of marine microbiota in the Red Sea, but the microbial communities recovered their original assemblage within one month following sandstorms, possibly due to the vast buffering capacity of the ocean. However, there exist significant concerns that the increased trend in dust activities due to global climate changes could prolong the recovery period or make irreversible modifications in microbial community composition, with major consequences on marine health.

## Methods

### Meteorological and atmospheric dust data

Meteorological data for the 2016 and 2017 sandstorm events (Table S1, Additional File 1) were obtained from the weather observatory at King Abdulaziz International Airport in Jeddah, Saudi Arabia. The PM_10_ conservative estimate in Table S1 (Additional File 1) was calculated according to D’Almeida’s correlation analysis: C_PM10_ = 914.06 × V^−0.73^ + 19.03, where C is the estimated concentration of PM_10_ in µgm^−3^ and V is the lowest daily visibility in km (Dalmeida 1986). The Satellite images of dust over the Red Sea in Fig. 2a-b were downloaded directly from the NASA MODIS website https://modis.gsfc.nasa.gov/data/ by providing the coordinates and dates. Atmospheric dust data generated by the SKIRON dust forecasting model was provided to us by the University of Athens (https://forecast.uoa.gr/en/forecast-maps/dust/central-asia). The National Oceanic and Atmospheric Administration (NOAA) HYSPLIT [55] back trajectory model was used to determine the origins of the 2016 and 2017 sandstorms based on the direction of air parcels arriving at the vertical height of 500 m, 1000 m, and 1500 m above ground level.

### Sample collection

Water samples for microbial analysis were collected from the Red Sea surface waters during the 2016 (June-Aug) and 2017 (Feb-Apr) sampling events (Table S1, Additional File 1), coinciding with two major sandstorms that originated from Nubian and Libyan Deserts, respectively. A total of four time points per year were chosen for comparison: 1) approximately one month prior to sandstorms as pre-sandstorm control; 2) approximately 2–3 days following sandstorms; 3) six days following sandstorms; 4) approximately one month after sandstorms as post-sandstorm control. For details on the specific sampling dates and the corresponding meteorological data, please refer to Table S1, Additional File 1. Samplings were conducted abroad a vessel using Niskin bottles, with CTD loggers that measured conductivity, temperature, depths, %O_2_ saturation, and pH. For each time point, 30L of seawater samples were collected, each from two shallow depths (1 m and 10 m) at two different locations (STN.A coordinates 22° 17.988’N, 39° 03.427’E and STN.B coordinates 22° 18.549’N, 39° 03.480’E), approximately one kilometer apart (Fig. 1), and transferred to 30L sterilized carboys. All samples were immediately transported to our laboratory in King Abdullah University of Science and Technology (KAUST), which is approximately 5 km away from the sampling points and were processed immediately for Chemical and microbiota analysis. One dust sample from the 2017 sandstorm event (March 20th) was also collected as follow. A sterile plastic sheet was secured on a tall table on the 5^th^ floor balcony of the CBRC building in KAUST. After 24 h, the dust sample on the sheet was collected and stored at -20 °C freezer for subsequent DNA extraction, as explained below.

### Chemical analysis

Metals (Ca, Mg, Na, S), trace metals (Fe, Mo, Mn, Zn, Cu), and nutrients (Si, NH_3_, PO_4_, NO_2_, NO_3_) in the Red Sea waters were analyzed and compared across different samples and time points (Table S4). The metal and trace metal analyses were done using Inductivity Coupled Plasma Mass Spectrometry. The dissolved organic matter (nutrients) analyses were done using Liquid Chromatography with Organic Carbon/Nitrogen Detection.

### Serial filtration of the Red Sea water samples for microbial analysis

The Red Sea water samples were serially filtered through 5micron, 0.8micron, and 0.22micron polycarbonate filters (47 mm diameter, Millipore, Bedford, MA, USA), and the membranes were immediately stored at -80 °C until the DNA extraction step. Serial filtration enabled examination of different populations of cells with: 1) microorganisms on the 5micron filters representing particle-bound, filamentous, and/or planktonic-associated microbial communities; 2) those on 0.8micron filters (between 5–0.8 micron) representing a combination of bound (as in 1, above) and free-living microbial communities; and 3) those on 0.22micron filters (between 0.8–0.22micron) representing the free-living microbial communities.

### DNA extraction

DNA was extracted from the frozen polycarbonate membranes using the phenol–chloroform extraction method described by Rusch et al. [54]. DNA was extracted from one gram of dust collected during the 2017 (March 20^th^) sandstorm using the Mo-Bio Power Soil DNA extraction kit (Qiagen, USA), as recommended by the manufacturer. DNA concentration was measured using Invitrogen Quant-iT Qubit dsDNA HS Assay Kit (Fisher Scientific, USA).

### The 16S rRNA amplification, metagenomic library preparation, and sequencing

The Illumina adaptors and PCR primer pairs targeting the V3 and V4 regions of the 16S rRNA gene (Table S5, Additional File 1) was ordered through Integrated DNA Technologies, Inc. Illinois, USA). The pair-end 16S rRNA gene amplification and metagenomic library preparation were conducted using the Illumina Nextera XT DNA Library Preparation Kit (UAE, ME), following Illumina protocols. Sequencing was conducted on an Illumina MiSeq platform (Illumina Inc.).

### Bioinformatics analysis

Bioinformatics analysis was conducted according to QIIME 16S rRNA Bacteria and Archaea standard operating procedure published by Langille's research lab in Dalhousie University in Canada [53]. In short, the 16S rRNA Illumina short reads were first quality inspected by FastQC, then each paired-end read was stitched together by PEAR. After merging, the stitched reads were filtered by a custom Perl script according to quality score and length. Potential chimeric sequences were removed from those qualified reads based on VSEARCH. OTUs were picked by the Open-reference strategy of QIIME, and singleton OTUs were removed. Following sample depth normalization (100,000 reads for each sample), alpha-diversity rarefaction and beta-diversity analyses were conducted with QIIME standard Python scripts. Final results were summarized and visualized by QIIME Python scripts, custom Perl scripts, and/or custom R scripts. The Greengenes database was used as a reference. The quality of the Illumina reads is summarized in Table S2, Additional File 1.

### Data and statistical analysis

For data and statistical analysis, samples from 2 different depths (1 m and 10 m) and 2 different stations (STN.A and STN.B) were treated as replicates, since they were all considered surface waters and had similar chemical (Table S4, Additional File 1) and microbial OTU profiles (Figs. 4–5); and the data throughout the paper were shown as the average of these replicates. Data were analyzed according to specific filter sizes (5, 0.8, and 0.22microns) to show changes in different size fractions. Turkey’s Honestly Significance Difference (HSD) test was used to examine if the observed changes in the average relative abundance of microbial community composition across different time points were statistically significant. All statistical tests were implemented with R multcomp package, and *P* values < 0.05 were considered significant.

## Supplementary Information

**Additional file 1.**

## Data Availability

Raw sequencing data generated in this study have been deposited in the DDBJ Sequence Read Archive (DRA) under accession number DRA013529 (Run ID: DRR350427-DRR350523). All data analyzed during this study are included in this article and its supplementary information files.

## Abbreviations

MODIS: Moderate Resolution Imaging Spectroradiometer
HYSPLIT: Hybrid Single-Particle Lagrangian Integrated Trajectory model
PM_10_: Particulate Matters with a diameter of 10 µm or less
OTU: Operational Taxonomic Unit
PCoA: Principal Coordinate Analysis
DOM: Dissolved Organic Matter
NOAA: National Oceanic and Atmospheric Administration
KAUST: King Abdullah University of Science and Technology
