# Supplementary material for: A cautionary signal from the Red Sea on the impact of increased dust activity on marine microbiota
Source: BMC Genomics. 2022 Apr 7;23:277. doi: 10.1186/s12864-022-08485-w (PMC8991508; doi:10.1186/s12864-022-08485-w)
Supplement: Supplementary file 1 — Additional file 1. [file 12864_2022_8485_MOESM1_ESM.pdf]

Supplemental Materials

A cautionary signal from the Red Sea on the impact of increased dust activity on marine microbiota.

Hayedeh Behzad, Hajime Ohyanagi, Badr Alharbi, Martin Ibarra, Mohammed Alarawi, Yoshimoto Saito, Carlos M. Duarte, Vladimir Bajic, Katsuhiko Mineta, Takashi Gojobori

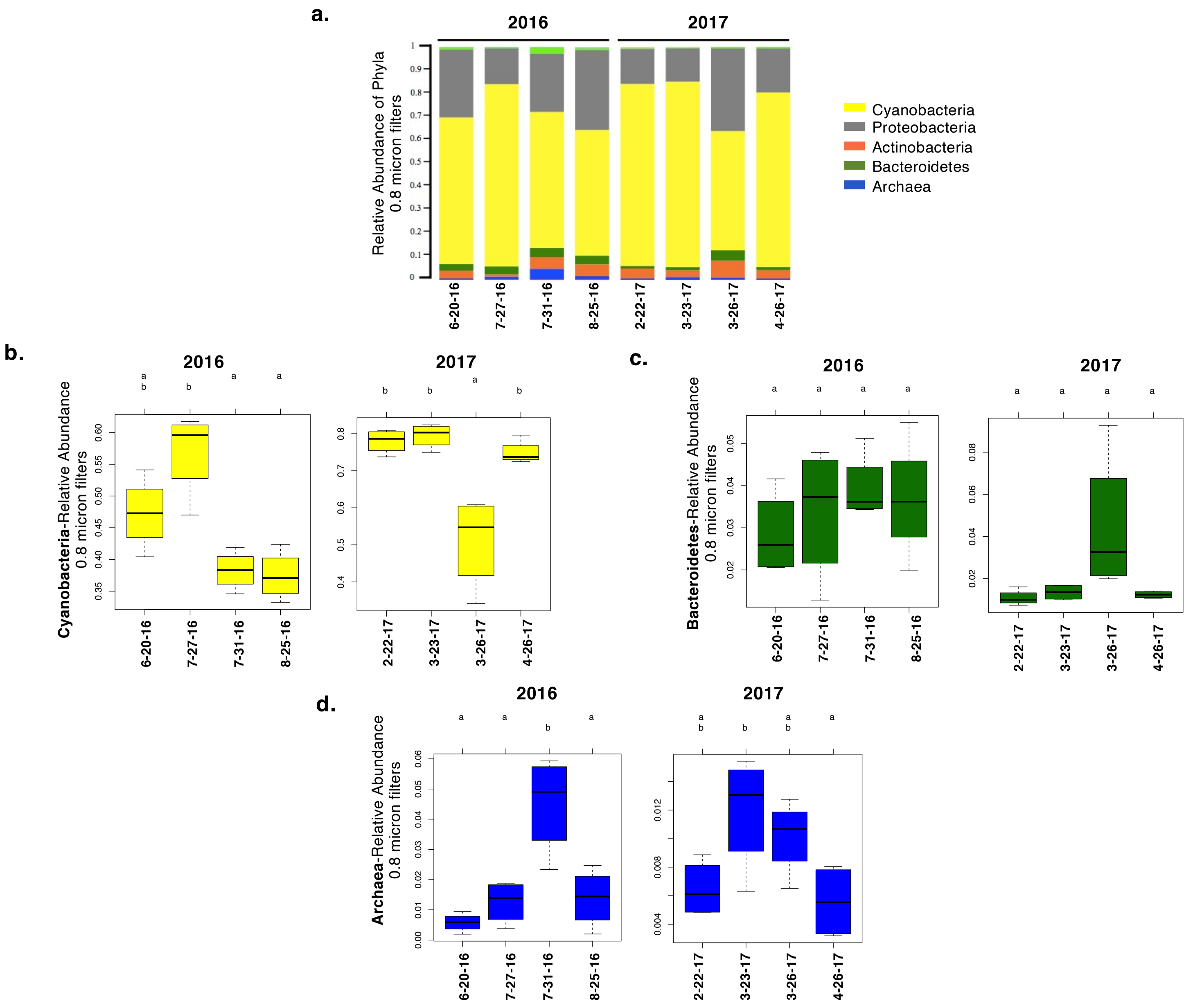

Figure S1. Taxonomic changes in microbial phyla in the Red Sea surface waters in response to the 2016 and 2017 sandstorm events. Graphs showing the changes in major phyla obtained from 0.8micron filters. For each time point, the data from 2 different depths (1m and 10m) at two different stations (STN.A and STN.B) were treated as replicates, the average of which were used for plotting graphs and statistical analysis. **a**, Bar graph shows changes in the average relative abundance of the most abundant Phyla during the 2016 (left) and 2017 (right) sampling events. Box plots demonstrate the average relative abundance of the most affected phyla, as follow: **b**, Cyanobacteria; **c**, Bacteroidetes; **d,** Archaea. Turkey's Honestly Significance Difference (HSD) test was used to analyze significant differences between time points, where different alphabetical letters on top of each graph denotes significant differences between the time points.

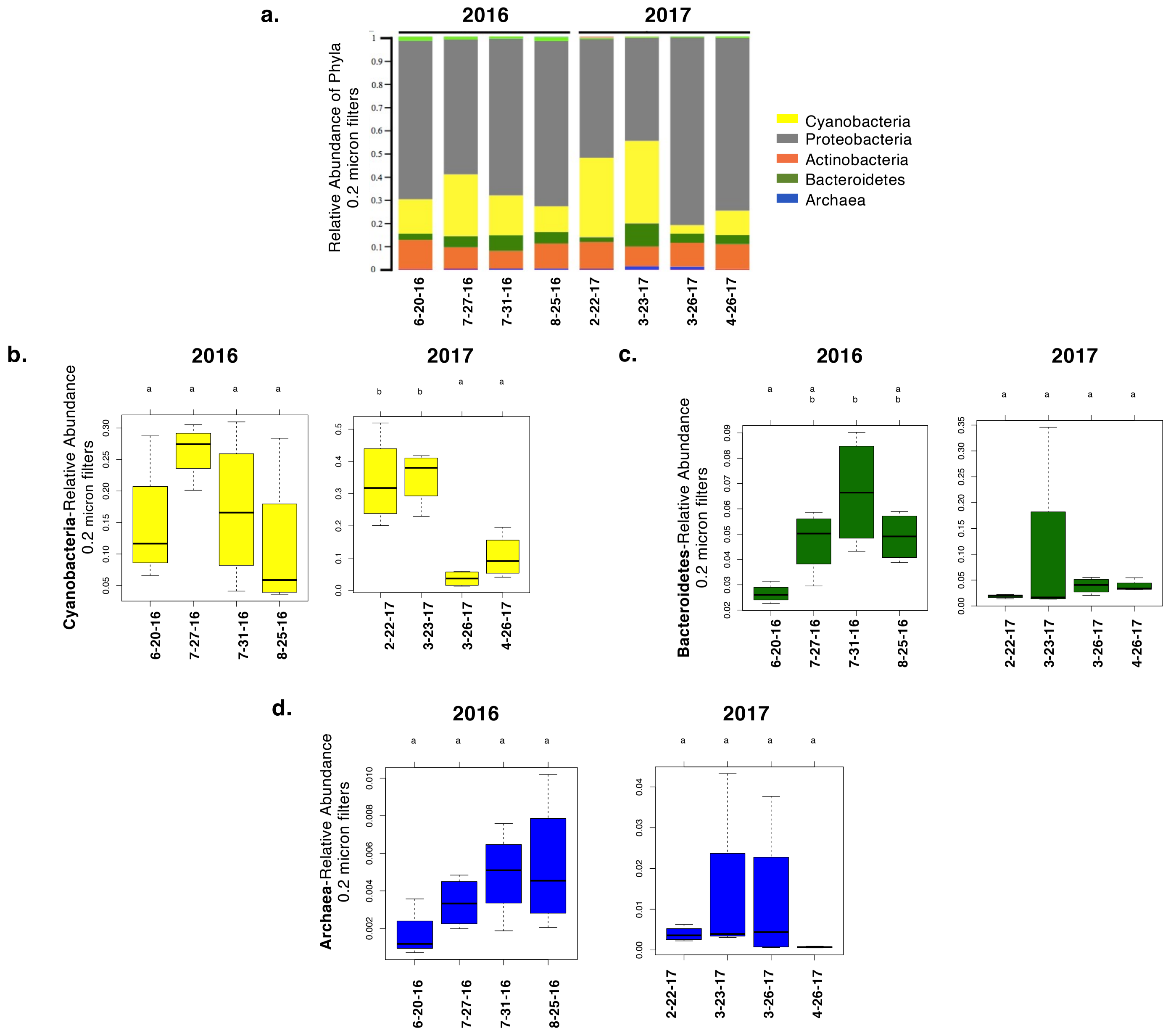

Figure S2. Taxonomic changes in microbial phyla in the Red Sea surface waters in response to the 2016 and 2017 sandstorms. Graphs showing the changes in major phyla on the 0.2micron filters. For each time point, the data from 2 different depths (1m and 10m) at two different stations (STN.A and STN.B) were treated as replicates, the average of which were used for plotting graphs and statistical analysis. **a,** Bar graph shows changes in the average relative abundance of the most abundant Phyla during the 2016 (left) and 2017 (right) sampling events. Box plots demonstrate the average relative abundance of the most affected phyla, as follow: **b**, Cyanobacteria; **c**, Bacteroidetes; **d**, Archaea. Turkey's Honestly Significance Difference (HSD) test was used to analyze significant differences between time points, where different alphabetical letters on top of each graph denotes significant differences between the time points.

Table S1. Environmental data obtained from the weather observatory at King Abdulaziz International Airport in Jeddah, Saudi Arabia.

Date	Visibility (km)	Duration of Visibility ≤ 3km (hour)	Estimated PM_{10} ($\mu g/m^3$)***	Wind direction	Mean wind speed (km/h)	Temp. (°C)	Humidity (%)
6-20-16*	≥ 10	0	189	N	15	35	38
7-25-16**	3-10	15	429	SSE	22	34.4	46
7-26-16**	2-8	8	570	WSW	10	35.9	37
7-27-16*	5-10	0	301	NNW	13	33.7	49
7-31-16*	≥ 10	0	189	SSW	11	33.6	57
8-25-16*	≥ 10	0	189	WSW	9	33.2	59
2-22-17*	≥ 10	0	189	NNW	13	26.3	60
3-19-17**	1-8	16	933	NNW	26	26.2	56
3-20-17**	1-8	17	933	N	19	24.2	51
3-23-17*	6-8	0	266	S	10	26.8	64
3-26-17*	≥ 10	0	189	NNW	10	23.8	62
4-26-17*	≥ 10	0	189	NNW	13	28.3	61

* Indicates sampling dates- the time points used.

** Indicates sandstorm dates- no sampling was conducted due to extreme weather conditions.

*** The estimates PM_{10} values were calculated according to D'Almeida's (Dalmeida 1986) correlation analysis using the following regression equation: $CPM_{10} = 914.06 \times V^{-0.73} + 19.03$, where CPM_{10} is the estimated concentration of PM_{10} and V is the lowest visibility in km.

Table S2. The 2016 (top row) and 2017 (bottom row) Illumina 16S rRNA amplicon reads: original, merged, and filtered/chimeras removed.

Year	Number of Read Pairs (Original)	Average Length Original (bp)	Number of Read Pairs (Merged)	Survival Rate (Merged/Original)	Average Length Merged (bp)	Number of Merged Reads (Filtered, Non-chimera)	Final Reads Average Length (bp)
2016	350,104 ± 17408	300 ± 0.005	344,373 ± 16,832	0.9856 ± 0.0012	444 ± 0.228	245,812 ± 9,838	444 ± 0.2201
2017	288,325 ± 10,306	300 ± 0.024	286,923 ± 10,271	0.9950 ± 0.0001	444 ± 0.251	222,601 ± 8,599	443 ± 0.2415

Data represents mean of all the samples per year ± Standard Error of the mean.

Table S3. Average richness and diversity of OTUs across different years (a) and different time points per year (b).

(a)

Year	Observed OTU	Chao-1	Shannon	Simpson
2016	840.56±13.15	1009.72±12.73	4.704±0.152	0.8006±0.020
2017	842.10±10.43	992.41±11.80	4.806±0.154	0.8174±0.019

Data in (**a**) and (**b**) represents mean of all the samples per year ± Standard Error of the mean.

(b)

Year	Observed OTU	Chao-1	Shannon	Simpson
6-20-16 (Pre)	890±20.525	1062±18.702	4.41±0.328	0.7508±0.046
7-27-16 (SD-1)	815±30.298	1014.808±20.850	4.206±0.387	0.7236±0.051
7-31-16 (SD-2)	774±15.008	934.931±17.786	4.923±0.209	0.8565±0.023
8-24-16 (Post)	874±25.580	1022±29.679	5.205±0.190	0.8638±0.023
2-22-17 (Pre)	826±23.995	965.922±28.6009	4.747±0.286	0.8240±0.031
3-23-17 (SD-1)	822±17.630	970.931±17.606	4.972±0.253	0.8521±0.024
3-26-17 (SD-2)	881±24.630	1031.877±28.052	5.307±0.217	0.8785±0.027
4-26-17 (Post)	834±13.287	996.532±15.042	4.255±0.397	0.7203±0.054

Pre (approx. one month before sandstorm); SD-1 (2-3 days post sandstorm); SD-2 (six days post sandstorm); Post (approx. one month after sandstorm)

Table S4: Metals, trace metals, and nutrients in the Red Sea Waters during the 2016 (top) and 2017 (bottom) sampling events

Dates	STN	Depth	Ca mg/L	Cu mg/L	Fe mg/L	Mg mg/L	Mn mg/L	Mo mg/L	P Mg/L	S mg/L	Zn mg/L	NH3 mg/L	Si mg/L	PO4 µg/L	NO2 µg/L	NO3 µg/L
20160620	STN.A	1m	448	ND	0.04	1503	ND	0.01	ND	1101	ND	5	35.5	ND	2.6	29.5
20160620	STN.A	10m	434	ND	0.04	1466	ND	0.01	ND	1074	ND	1.5	35.4	ND	ND	23.9
20160620	STN.B	1m	430	ND	0.03	1516	ND	0.01	ND	1113	ND	2.1	31.8	ND	ND	18.2
20160620	STN.B	10m	441	ND	0.03	1498	ND	0.01	ND	1096	ND	2.4	35.5	ND	ND	18.6
20160727	STN.A	1m	302	ND	0.04	1083	ND	0.01	ND	740	ND	1.8	58.5	ND	ND	33.7
20160727	STN.A	10m	378	ND	0.04	1346	ND	0.01	ND	923	ND	2.3	50.9	ND	ND	26.6
20160727	STN.B	1m	373	ND	0.03	1329	ND	0.01	ND	909	ND	1.8	50.5	ND	ND	20.6
20160727	STN.B	10m	370	ND	0.03	1304	ND	0.01	ND	892	ND	1	39.9	ND	ND	14.5
20160731	STN.A	1m	368	ND	0.03	967	ND	0.01	ND	705	ND	6.6	55.5	ND	1.0	25.3
20160731	STN.A	10m	372	ND	0.03	1207	ND	0.01	ND	861	ND	3	54.1	ND	ND	20.9
20160731	STN.B	1m	373	ND	0.04	1238	ND	0.01	ND	872	ND	3.5	54.9	ND	5.5	11.1
20160731	STN.B	10m	367	ND	0.03	1223	ND	0.01	ND	858	ND	6.7	51.6	ND	ND	9.7
20160824	STN.A	1m	397	ND	0.03	1378	ND	0.01	ND	947	ND	4.4	43.9	2.0	ND	21.1
20160824	STN.A	10m	409	ND	0.03	1336	ND	0.01	ND	918	ND	2.1	42.2	1.9	ND	16.7
20160824	STN.B	1m	402	ND	0.03	1331	ND	0.01	ND	919	ND	3.1	39.6	2.0	1.5	17.9
20160824	STN.B	10m	403	ND	0.03	1328	ND	0.01	ND	918	ND	2.6	43.2	1.5	ND	ND

Dates	STN	Depth	Ca mg/L	Cu mg/L	Fe mg/L	Mg mg/L	Mn mg/L	Mo mg/L	P Mg/L	S mg/L	Zn mg/L	NH3 mg/L	Si mg/L	PO4 µg/L	NO2 µg/L	NO3 µg/L
20170222	STN.A	1m	455.45	0.002	0	1486.07	ND	0.01	0.022	1071.5	0	17.57	47.2	4.038	ND	ND
20170222	STN.A	10m	456.23	0.002	0	1488.07	ND	0.01	0.025	1069.3	0	16.65	45	3.85	ND	ND
20170222	STN.B	10m	457.39	0.002	0.001	1497.43	ND	0.007	0.042	1063.7	0.005	32.41	45.1	3.028	ND	ND
20170222	STN.B	1m	463.17	0.002	0.001	1489	ND	0.008	0.049	1077.7	0.005	18.39	46.7	4.17	ND	ND
20170222	STN.A	10m	458.28	0.001	0.001	1505.29	ND	0.01	0.059	1078.9	0.006	17.5	48.8	3.441	ND	ND
20170323	STN.A	1m	456.04	0.002	0.002	1479.71	ND	0.011	0.025	1075.3	0.006	6.7	52.9	6.989	ND	ND
20170323	STN.B	10m	463.92	0.002	0.001	1483.47	ND	0.007	0.039	1098.3	0.006	4.0	53.2	3.06	ND	ND
20170323	STN.B	1m	465.06	0.002	0.003	1484.61	ND	0.007	0.054	1102.1	0	6.6	56.5	2.584	ND	ND
20170323	STN.A	10m	463.22	0.002	0.002	1487.91	ND	0.007	0.032	1094.8	0.006	5.9	49.7	3.07	ND	ND
20170326	STN.A	1m	465.25	0.003	0.002	1517.8	ND	0.009	0.048	1103.1	0.006	3.0	51.1	2.133	ND	ND
20170326	STN.B	10m	466.35	0.002	0.001	1494.31	ND	0.012	0.032	1102.7	0.005	5.6	53.1	2.296	ND	ND
20170326	STN.B	1m	468.09	0.002	0.002	1508.1	ND	0.009	0.072	1109.3	0.005	6.3	43.8	1.279	ND	ND
20170326	STN.A	10m	533.69	0.002	0.001	1720.66	ND	0.011	0.041	1264.1	0.003	5.0	54.3	2.681	ND	ND
20170426	STN.A	10m	551.69	0.003	0.001	1818.93	ND	0.008	0.04	1298.8	0.005	0.5	50.9	1.765	ND	ND
20170426	STN.B	1m	497.46	0.001	0.001	1630.47	ND	0.008	0.045	1171.5	0.004	0.1	52.3	1.053	ND	ND
20170426	STN.B	10m	455.71	0.002	0.001	1490.08	ND	0.007	0.051	1077.7	0	1.3	55.9	3.018	ND	ND

Table S5. 16S rRNA PCR forward (top) and reverse (bottom) primers targeting the v3 and v4 region of 16S rRNA gene

16S rRNA PCR Forward Primer
5’ **TCGTCGGCAGCGTCAGATGTGTATAAGAGACAG**CCTACGGGNGGCWGCAG 3’
16S rRNA PCR Reverse Primer
5’ **GTCTCGTGGGCTCGGAGATGTGTATAAGAGACAG**GACTACHVGGGTATCTAATCC 3’

The bold fonts are the Illumina adaptor sequences.
